# Prevalence and association of type I IFN autoantibodies with clinical outcomes in critically ill Brazilian COVID-19 patients

**DOI:** 10.70962/jhi.20250031

**Published:** 2025-07-08

**Authors:** Graziela P Peduti, Isadora M Paiva, Adrian Gervais, Lorena V Andrade, Beatriz Vaconcelos, Pablo RS Oliveira, Luydson R Vasconcelos, Ricardo Khouri, Thiago M Cunha, Carlos DF Souza, Anderson C Armstrong, Jean-Laurent Casanova, Rodrigo F Carmo

**Affiliations:** 1 Postgraduate program in Biosciences, Federal University of São Francisco Valley, Petrolina, Brazil.; 2 Ribeirão Preto Medical School, University of São Paulo, Ribeirão Preto, Brazil.; 3 Laboratory of Human Genetics of Infectious Diseases, Necker Branch, INSERM U1163, Necker Hospital for Sick Children, Paris, France.; 4 University Paris Cité, Imagine Institute, Paris, France.; 5 Gonçalo Moniz Institute, Oswaldo Cruz Foundation, Salvador, Brazil.; 6 Biology Institute, Federal University of Bahia, Salvador, Brazil.; 7 Aggeu Magalhães Institute, Oswaldo Cruz Foundation, Recife, Brazil.; 8 College of Medicine, Federal University of São Francisco Valley, Petrolina, Brazil.; 9 St. Giles Laboratory of Human Genetics of Infectious Diseases, Rockefeller Branch, The Rockefeller University, New York, NY, USA.; 10 Howard Hughes Medical Institute, New York, NY, USA.; 11 Dept. of Pediatrics, Necker Hospital for Sick Children, AP-HP, Paris, France.

**Keywords:** cytokine, immunopathogenesis, interferon, SARS-CoV-2

## Abstract

Anti-type I interferon (IFN) autoantibodies (auto-Abs) impair antiviral immunity and have been associated with critical COVID-19. However, their prevalence and impact in Latin American populations remain incompletely delineated. This study assessed the prevalence of auto-Abs neutralizing 10 ng/mL IFN-α2 and IFN-ω in critically ill COVID-19 patients from a Brazilian cohort and their association with clinical outcomes. Among 209 patients, 14 (6.7%) tested positive, including 9.4% of deceased individuals. The proportion was 13.2% in patients older than 65 years. These individuals exhibited increased MIP-1β levels, altered hematological parameters, and low IFN-α levels. Survival analysis indicated shorter hospitalization survival times in auto-Ab-positive patients (log-rank = 0.01). The prevalence aligns with global reports, predominantly affecting older individuals. Our findings highlight the association between type I IFN auto-Abs and worse clinical outcomes, emphasizing the need for further studies to understand their biological role and clinical implications.

## Summary

Type I IFN autoantibodies (auto-Abs) impair antiviral immunity and are associated with severe COVID-19. This study reveals a 6.7% prevalence of auto-Abs in critically ill Brazilian patients, predominantly affecting older individuals, and links their presence to worse clinical outcomes.

## Introduction

Severe acute respiratory syndrome coronavirus 2 (SARS-CoV-2), the triggering agent of COVID-19, has led to unprecedented global health challenges. While most infections result in mild or moderate disease, a subset of individuals develops severe complications, often characterized by acute respiratory distress syndrome, systemic inflammation, and multi-organ failure (1). These outcomes are particularly prevalent among critically ill patients, suggesting that host-specific factors, such as immune dysregulation, play a pivotal role in disease progression (2).

Type I interferons (IFNs), first described in 1957 (3), are central cytokines in antiviral immunity. They serve as the first line of defense by limiting viral replication and modulating downstream immune responses (4). Inborn errors in genes of the type I IFNs pathways have been implicated in a wide range of severe viral diseases (5–11). In the context of COVID-19, a landmark study in 2020 by the international consortium COVID Human Genetic Effort revealed that at least 3.5% of patients with life-threatening COVID-19 pneumonia had known or novel genetic defects in loci involved in the TLR3- and TLR7-dependent induction and amplification of type I IFNs (8, 12). Subsequent studies demonstrated that at least 10% of patients with life-threatening COVID-19 pneumonia had autoantibodies (auto-Abs) neutralizing 10 ng/mL type I IFNs (13). Moreover, auto-Abs neutralizing 100 pg/mL accounted for 15% of cases and 20% of critical COVID-19 pneumonia cases in patients over 80 years old and a similar proportion of COVID-19-related deaths (14, 15).

Auto-Abs typically neutralize IFN-α, IFN-ω, or, less frequently, IFN-β (11). Those targeting IFN-α2 often neutralize all other 11 subtypes of IFN-α, potentially leading to heightened susceptibility to severe disease in patients with COVID-19. Even auto-Abs that exclusively neutralize IFN-ω are associated with increased disease severity (11, 13, 15). These pre-existing auto-Abs are a leading cause of critical COVID-19, surpassed only by age as a risk factor (14).

Although studies in North-American, European and Asian populations have reported auto-Abs prevalence rates of up to 18% in critically ill patients (16, 17, 26–35, 18, 36–45, 19, 46, 47, 20–25), data on their frequency and clinical impact in Latin American populations remain scarce (48, 49). In addition, the results in the literature are still contradictory regarding the role of auto-Abs against type I IFN in the clinical course of COVID-19 and their correlation with laboratory markers (17, 18, 28, 29).

In this study, we investigated the prevalence of auto-Abs neutralizing type I IFNs in critically ill patients with COVID-19 hospitalized in the Northeast Region of Brazil. The population of this region is characterized by significant genetic admixture of people from European, African, and Native American ancestry (50). Additionally, we explored their association with clinical and laboratory parameters to provide insights into their potential role in disease severity and outcomes.

## Results

**Figure 1.**
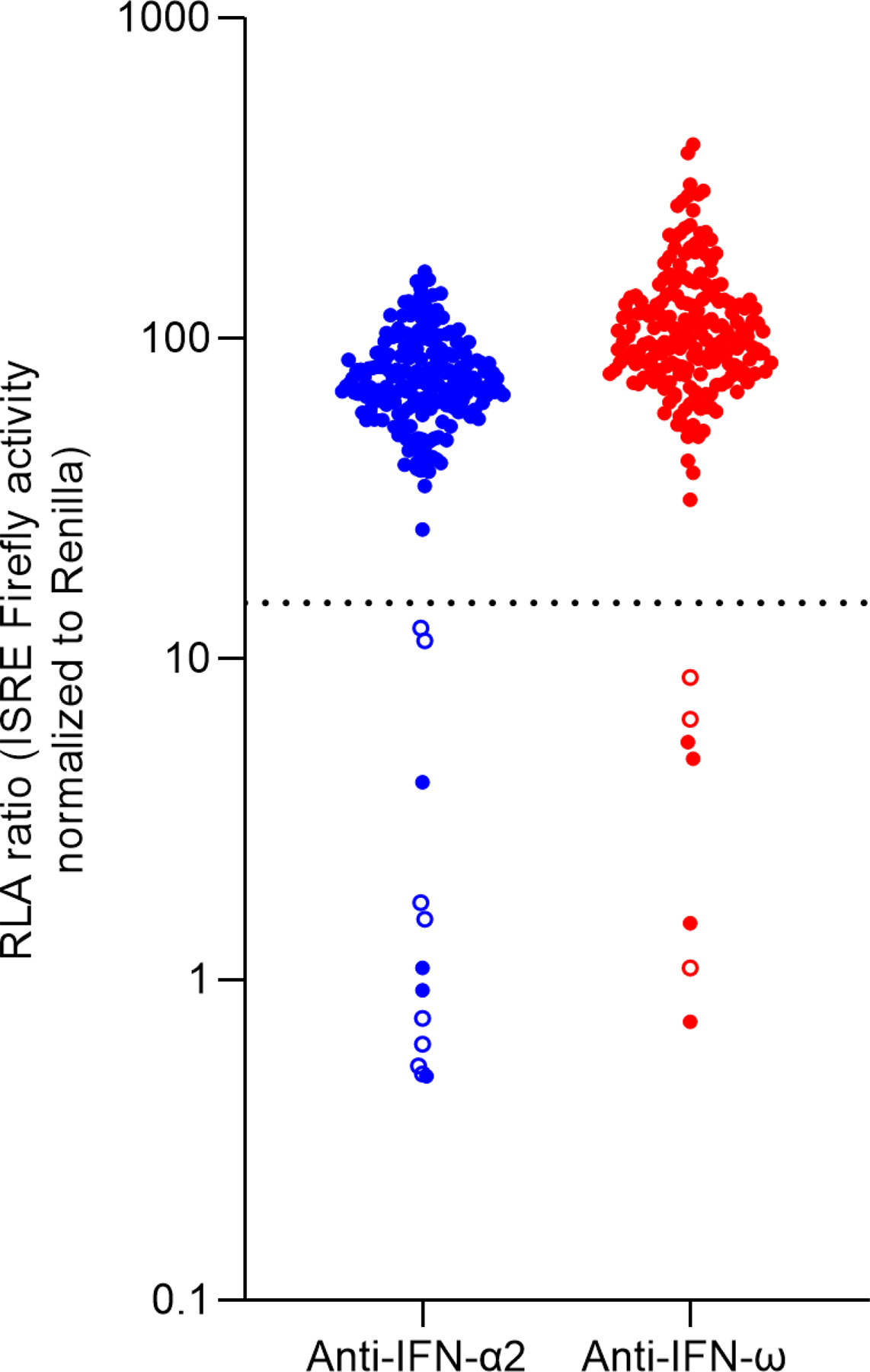
Neutralizing autoantibodies against IFN-α2 and/or IFN-ω in critically ill patients with COVID-19. RLA is shown (ISRE dual luciferase activity, with normalization against Renilla luciferase activity) after stimulation with 10 ng/ml of IFN-α2 or IFN-ω in the presence of plasma samples (n = 209). IFN: interferon; ISRE: interferon-stimulated response element; RLA: relative luciferase activity. All samples were tested twice independently. Empty circles indicate patients who have died. The dotted line indicates the cut-off.

A total of 209 critically ill patients were analyzed, with 85 (40.8%) dying during the course of the study. Regarding the presence of anti-type I IFN auto-Abs, 14 patients (6.7%) tested positive for anti-IFN-α2 and/or anti-IFN-ω auto-Abs. This proportion was even higher among individuals older than 65 years old (13.2%), compared to those under 65 years old (3.5%). Among those who tested positive, 11 (78.6%) had anti-IFN-α2 auto-Abs, and 7 (50.0%) had anti-IFN-ω auto-Abs. Specifically, 7 (50.0%) patients were positive exclusively for IFN-α2; 3 (21.4%) were positive only for IFN-ω auto-Abs, and 4 (28.57%) tested positive for both. Overall, 8 of 85 (9.4%) deceased patients carried the auto-Abs (Figure 1).

As described in Table 1, patients who tested positive for type I IFN auto-Abs were significantly older than those who tested negative (75 years versus 56 years, respectively; p < 0.001). No significant difference in sex distribution was observed between the groups (p = 0.461). Regarding preexisting comorbidities, a higher frequency of chronic heart disease was observed among patients positive for type I IFN auto-Abs (28.5% versus 3.5%, p < 0.001).

Table 2 displays the distribution of laboratory data obtained within 24 hours of hospital admission. Patients positive for type I IFN auto-Abs showed a trend to have higher levels of total leukocytes, neutrophils, and creatinine. However, these differences did not reach statistical significance (p = 0.136, p = 0.086, and p = 0.106, respectively).

**Figure 2.**
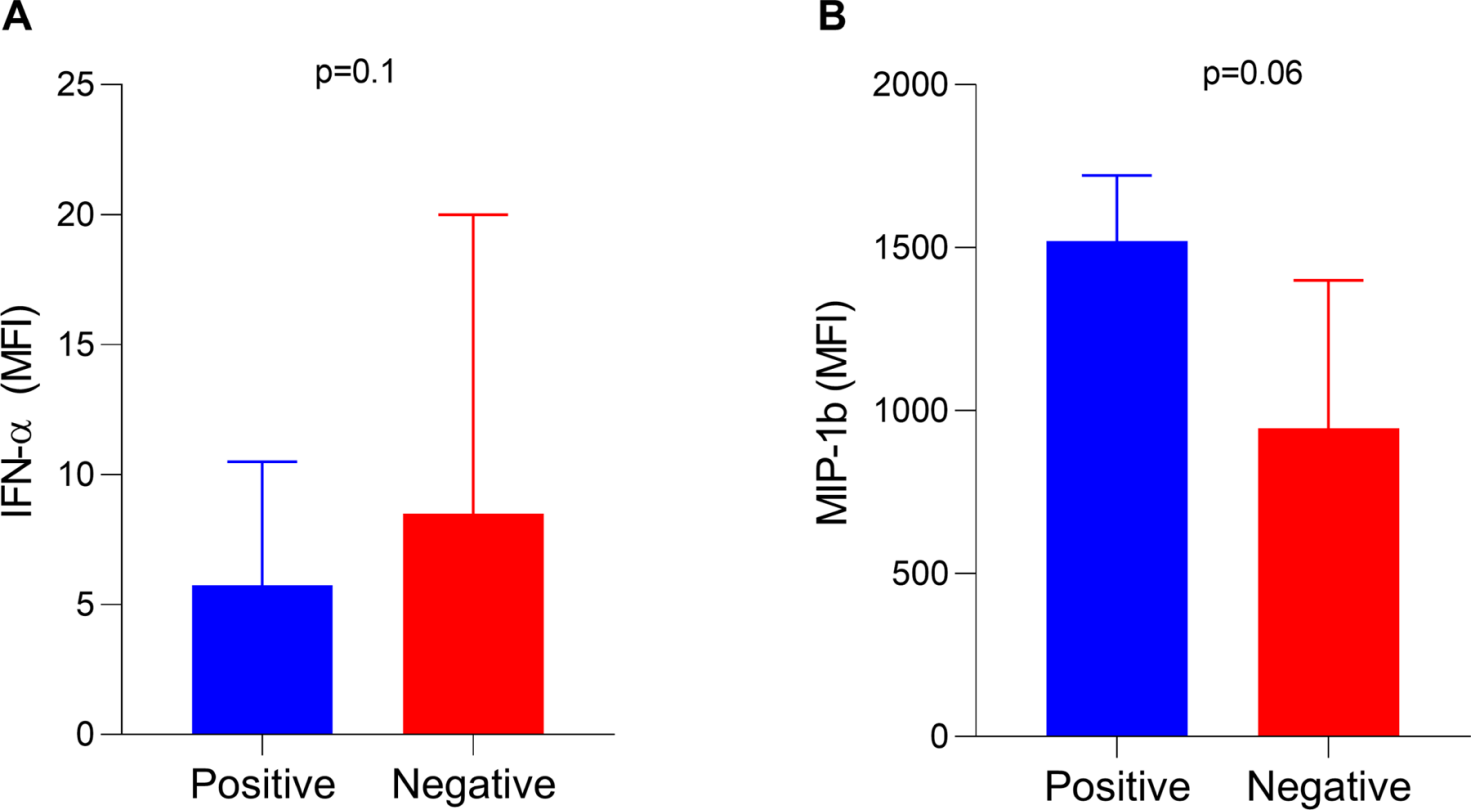
Serum levels of (A) IFN-α and (B) MIP-1β among patients positive or negative for autoantibodies against type I IFNs. Data are shown as median with interquartile range. The Mann–Whitney test was used to compare the groups. IFN: interferon; MFI: mean fluorescence intensity.

In addition to routine laboratory tests, we evaluated if the presence of type I anti-IFN auto-Abs is associated with serum levels of inflammatory markers in the first 24 hours of hospitalization. This analysis included 193 patients who had available data (14 patients positive for auto-Abs and 179 patients negative for auto-Abs). As expected, serum IFN-α levels were lower in patients positive for type I anti-IFN auto-Abs compared to those negative for these auto-Abs (5.75 MFI versus 8.50 MFI, respectively; p = 0.103) (Figure 2A). Conversely, patients positive for auto-Abs showed higher levels of MIP-1β (CCL4) compared to those negative for auto-Abs, with a borderline p-value (1,521 MFI versus 946 MFI, p =0.06) (Figure 2B). The other molecules investigated showed no significant differences between the groups (Supplementary Figure S1).

When evaluating the length of hospital stay, complications during hospitalization, and clinical outcomes, we observed that, although patients positive for type I IFN auto-Abs had shorter hospital stays, they experienced worse clinical courses (Table 3). These patients had higher rates of requiring ventilatory support, hemodialysis, sepsis, shock, myocardial infarction, and death, although none of these differences were statistically significant.

**Figure 3.**
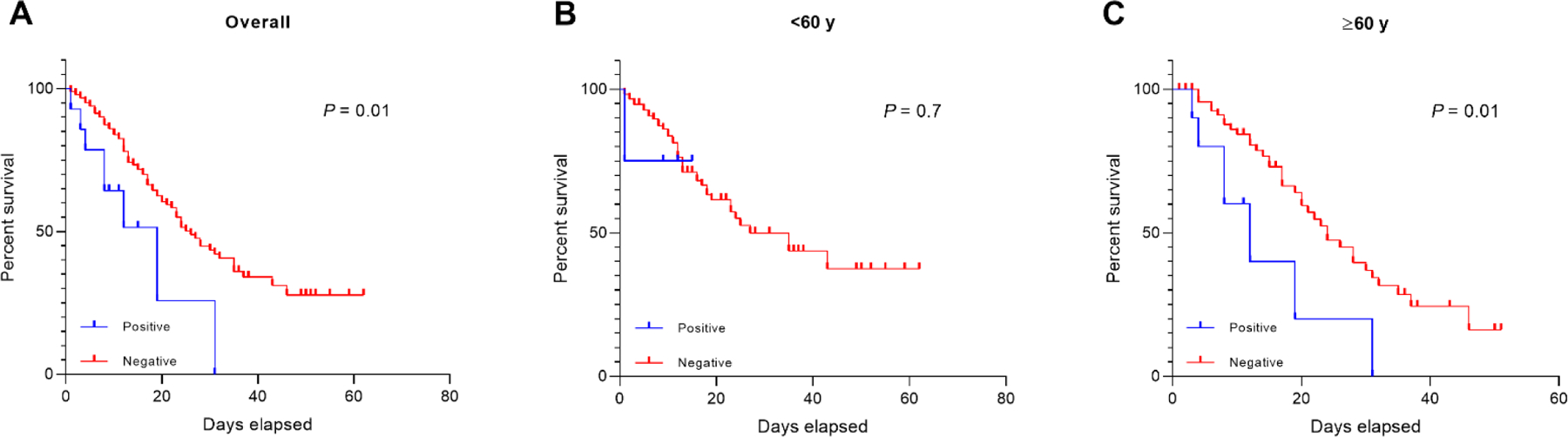
Impact of type I IFN autoantibodies on mortality risk in critically ill COVID-19 patients, analyzed using the Kaplan–Meier method and stratified by age. (A) overall subjects (B) <60 years old and (C) ≥60 years old.

To assess whether the presence of autoantibodies (auto-Abs) against type I interferons (IFNs) influences survival time after hospitalization, we constructed Kaplan– Meier survival curves. Patients positive for neutralizing auto-Abs had a significantly shorter survival time compared to negative patients (median survival time in days: auto-Abs positive, 19; auto-Abs negative, 26; log-rank = 0.01) (Figure 3A). Given that age is a known risk factor for COVID-19 mortality, we stratified the survival analysis by age group (<60 vs. ≥60 years). Among individuals aged ≥60 years, those with auto-Abs had a markedly shorter survival time than those without (median survival time in days: auto-Abs positive, 12; auto-Abs negative, 24; log-rank = 0.01). In contrast, no significant difference was observed in individuals under 60 years of age (Figure 3B,C).

To further evaluate the independent impact of auto-Abs on survival, we performed a multivariate Cox proportional hazards regression analysis, including age and chronic heart disease as covariates. The results revealed that age (HR: 1.01, 95% CI: 1.00–1.02, p = 0.042) and chronic heart disease (HR: 3.47, 95% CI: 1.51–7.94, p = 0.003) were significantly associated with reduced survival. However, after adjustment, the presence of auto-Abs did not remain significantly associated with mortality (HR: 1.47, 95% CI: 0.65–3.33, p = 0.350), suggesting that age and chronic heart disease are stronger predictors of mortality in this cohort (Table S1).

## Discussion

In this study, we demonstrated, for the first time in a cohort of Brazilian adults, a prevalence of 6.7% of IFN-α2 and/or IFN-ω neutralizing auto-Abs (10 ng/mL) in critically ill individuals hospitalized with COVID-19. The proportion is even higher among deceased patients (9.4%) and among patients older than 65 years (13.2%). These individuals were significantly older and had a higher prevalence of chronic heart disease compared to those without auto-Abs. In addition, positive patients had a shorter survival time after hospitalization.

We demonstrate that the prevalence of type I IFN auto-Abs in Latin American patients with critical COVID-19 is similar to that reported in European and Asian populations, ranging from 7.9% to 18% (16, 17, 26–35, 18, 36–45, 19, 46, 47, 20–25). Studies in Latin American populations are scarce. A Colombian study (48) and a Peruvian study (49), using a commercial ELISA kit, reported a prevalence of auto-Abs against IFN-α in individuals with severe COVID-19, at 16.7% and 48.2%, respectively.

It is important to note that these values may vary across studies depending on the detection methods employed and the severity criteria used. In this study, we utilized a luciferase reporter gene assay with a concentration of 10 ng/mL of IFN, chosen due to its higher sensitivity in detecting clinically relevant neutralizing activity, especially in critically ill patients, as previously reported (15). Studies using Gyros or ELISA tend to yield higher positivity rates (22, 24, 28). Gonçalves et al. (2021) observed a 25% positivity rate in critical COVID-19 patients using ELISA, while only 18% exhibited neutralizing activity through the luciferase reporter assay at a concentration of 10 ng/mL of IFN-α2 or IFN-ω (24). Similarly, Solanich et al. (2021) reported a 17.8% positivity rate in critical patients using ELISA, compared to 9.5% using the luciferase neutralization assay at a concentration of 10 ng/mL of IFN-α2 or IFN-ω (28). Additionally, a French study evaluating critical patients with COVID-19 found significant variability in prevalence when using Gyros, ELISA, and luciferase neutralization assays, reporting positivity rates of 77%, 6.5%, and 7.9%, respectively. Notably, 2.9% of patients had auto-Abs neutralizing 10 ng/mL of IFN-α2 and/or IFN-ω, while 5% had auto-Abs neutralizing only lower concentrations (100 pg/mL) of IFN-α2 or IFN-ω (22). Therefore, severity criteria, as well as the methods used to detect these autoantibodies, may explain the variation in the positivity rate across studies.

We observed that individuals positive for type I IFN auto-Abs were significantly older and had a higher prevalence of chronic heart disease as an underlying condition. The association between the presence of auto-Abs and age has been well documented in the literature (14, 15, 51, 52). Consistent with this, Bastard et al. (2021) reported that the prevalence of neutralizing auto-Abs against type I IFNs, excluding IFN-β, increases significantly with age in the general population, reaching over 4% in individuals older than 70 years. Furthermore, these auto-Abs accounted for approximately 20% of critical COVID-19 pneumonia cases in patients over 80 years old and around 20% of total COVID-19-related deaths in a cohort of 3,595 individuals from diverse ethnic backgrounds (15). Conversely, other studies have failed to demonstrate a relationship between the presence of anti-IFN auto-Abs and age (17, 18, 20, 24, 28, 29). The reason for these conflicting results is not entirely clear, but it may be related to differences in the demographic composition of study populations. For instance, our study included individuals aged 19 to 93 years, with a predominance of individuals between 45 and 70 years, a composition similar to that of the 2021 study by Bastard et al. In contrast, other studies primarily included older individuals with a shorter age range. These demographic differences may also explain the higher prevalence of chronic heart disease among auto-Abs-positive individuals observed in our study, which diverges from the findings of previous research. Further studies are needed to explore the relationship between these conditions.

We evaluated whether routine laboratory tests collected within 24 hours of hospital admission were associated with the presence of type I IFN auto-Abs. Although statistical significance was not achieved, we observed a trend indicating that patients with type I IFN auto-Abs had higher total leukocyte and neutrophil counts compared to those without auto-Abs. Previous studies have demonstrated that critically ill patients with type I IFN auto-Abs exhibit elevated levels of certain laboratory parameters upon admission, including leukocytes, neutrophils, and C-reactive protein (17, 18, 28, 29). These findings likely reflect an exaggerated inflammatory response driven by impaired IFN signaling. Type I IFNs play a crucial role in the early antiviral response, promoting viral clearance and modulating inflammation (4). The presence of neutralizing auto-Abs against type I IFNs disrupts these protective mechanisms, resulting in uncontrolled viral replication and the activation of secondary immune pathways (16, 30, 53). This dysregulation can lead to heightened systemic inflammation, as evidenced by increased levels of leukocytes, neutrophils, and C-reactive protein.

To better elucidate the relationship between the presence of auto-Abs and increased systemic inflammation, we quantified serum levels of 21 immune molecules, including cytokines and chemokines. While no significant differences were observed across most analyzed molecules, patients positive for auto-Abs exhibited decreased levels of IFN-α, consistent with previous findings (13, 17, 24). Type I interferons, particularly IFN-α, are critical for the initial antiviral immune response. They induce the expression of IFN-stimulated genes, which play a pivotal role in limiting viral replication and coordinating downstream immune responses (4). Studies have demonstrated that neutralizing auto-Abs can infiltrate both the upper and lower respiratory tracts, effectively blocking the induction of IFN-stimulated genes and compromising these protective mechanisms (27, 53). This disruption likely facilitates uncontrolled viral replication, undermining an effective antiviral response and contributing to the heightened inflammatory state observed in severe cases.

Additionally, a borderline p value was observed in the levels of MIP-1β (CCL4), with individuals positive for auto-Abs displaying higher levels compared to negative patients. MIP-1β is a chemokine predominantly secreted by monocytes, macrophages, and T cells in response to inflammatory stimuli (54). The observed increase in MIP-1β levels may represent a compensatory immune response to the impaired antiviral activity resulting from reduced type I IFN levels. However, elevated MIP-1β levels could also exacerbate systemic inflammation and tissue damage, as suggested by the concurrent increases in hematological parameters and acute-phase markers in the patients with neutralizing auto-Abs. Although studies investigating the role of MIP-1β in COVID-19 are limited, existing evidence suggests that its levels are elevated in critically ill patients (55–58). Our findings are the first to establish a potential relationship between MIP-1β and the presence of auto-Abs against type I IFNs, highlighting the need for further research to understand its role in the pathophysiology of severe COVID-19.

In line with this, patients positive for type I IFN auto-Abs tended to experience more severe complications, including a higher requirement for invasive ventilatory support, hemodialysis, and increased mortality rates. An Italian study of hospitalized patients with COVID-19 similarly reported that the presence of anti-IFN auto-Abs was associated with an elevated risk of intensive care unit admission and delayed viral clearance (16). Likewise, Solanich et al. (2021) demonstrated that critically ill COVID-19 patients with type I IFN auto-Abs had a higher likelihood of developing acute kidney injury during hospitalization (28).

Furthermore, a Spanish study found that patients with neutralizing auto-Abs required greater oxygen support and showed a trend toward higher mortality risk (29). In a French cohort, the presence of neutralizing auto-Abs was linked to increased mortality, with auto-Abs detected in 21% of patients who died from COVID-19 pneumonia (22). A European study spanning multiple centers in Germany and Switzerland revealed that patients positive for neutralizing anti-IFN auto-Abs had higher severity scores; greater reliance on invasive mechanical ventilation, renal replacement therapy, and/or ECMO; increased mortality risk; and shorter survival times (17).

To assess the independent impact of type I IFN auto-Abs on survival, we performed a multivariate Cox proportional hazards regression analysis, including age and chronic heart disease as covariates. The results revealed that age (HR: 1.014, 95% CI: 1.001–1.028, p = 0.042) and chronic heart disease (HR: 3.473, 95% CI: 1.519–7.940, p = 0.003) were significantly associated with reduced survival. However, after adjusting for these covariates, the presence of auto-Abs did not remain significantly associated with mortality (HR: 1.476, 95% CI: 0.652–3.339, p = 0.350). These findings suggest that the previously observed association between auto-Abs and reduced survival may be partially explained by the higher prevalence of age and comorbidities in the auto-Ab positive group. However, given the limited sample size and the small number of auto-Ab positive patients, the analysis may have been underpowered to detect a significant association, indicating a potential for a Type II error.

Although some studies have not identified an association between auto-Abs and clinical complications (18, 20, 24, 27), the majority of evidence supports the hypothesis that the neutralization of type I IFN activity compromises both viral clearance and the early antiviral defense. This disruption likely exacerbates systemic inflammation, promotes organ failure, and increases susceptibility to severe COVID-19 complications.

This study has several limitations, including a small sample size, a single-center design, and the lack of experiments using a lower concentration of IFN (100 pg/mL), which could have provided additional insights into the presence of auto-Abs with weaker neutralizing activity. Therefore, larger, multicenter studies are needed to better evaluate the relationship between autoantibodies and COVID-19 outcomes. Nonetheless, to the best of our knowledge, it represents the largest cohort of Latin American patients assessed for the prevalence of type I IFN auto-Abs in critically ill COVID-19 cases.

Our findings indicate that the prevalence of type I IFN auto-Abs in critically ill patients from Brazil aligns with reports from other regions worldwide, predominantly affecting older individuals. Moreover, we observed that individuals positive for these auto-Abs exhibited increased levels of MIP-1β, altered hematological parameters, and suppressed IFN-α levels. These findings may partially explain the observed trend toward worse clinical outcomes during hospitalization. Future studies should further investigate the relationship between anti-IFN auto-Abs, inflammatory markers, and clinical parameters in order to better understand how these auto-Abs influence disease progression and identify potential therapeutic strategies to mitigate their impact.

## Materials and methods

### Ethical aspects

This study obtained ethical approval from the Ethics Committee of the Hospital das Clínicas of the Federal University of Pernambuco (HC/UFPE, acronym in Portuguese), under registration number CAAE: 36613520.0.0000.5640. The research was conducted in accordance with the provisions of the Declaration of Helsinki and the Good Clinical Practice guidelines. Written informed consent was obtained from all participants or carers.

### Study population

This study included critically ill patients aged 18 years or older, with a confirmed diagnosis of COVID-19 through RT-qPCR or serological testing, recruited from March 2020 to July 2021 at the University Hospital of the Federal University of Vale do São Francisco, Brazil. Patients were classified as critical when they developed critical disease, including pulmonary symptoms requiring high-flow oxygen therapy or mechanical ventilation (continuous positive airway pressure, bilevel positive airway pressure, and intubation), septic shock, or damage to any other organ that required intensive care unit admission.

### Sample collection and processing

Blood samples were collected by venipuncture, within 24 hours of hospital admission. After collection, the samples were centrifuged at 1500 RPM for 10 minutes to obtain serum and plasma. The samples were then transferred to cryogenic tubes (Greiner Bio-One) and stored at −80 °C.

### Data collection

Data from patients were collected by reviewing electronic medical records, focusing on laboratory results, clinical details, and demographic information. Each patient was assigned a unique internal identification number, and their data were organized in an electronic spreadsheet for analysis.

### Detection of type I IFN autoantibodies

The blocking activity of anti-IFN-α2 and anti-IFN-ω auto-Abs was assessed by measuring luciferase reporter activity as described by Bastard et al. (2021) (15). Briefly, HEK293T cells were transfected with a plasmid containing the firefly luciferase gene under the control of the human ISRE promoter in the pGL4.45 backbone and a plasmid constitutively expressing Renilla luciferase for normalization (pRL-SV40). Cells were transfected in the presence of Lipofectamine 3000™ transfection reagent (reference number L3000015; Invitrogen) for 16 hours. Cells in DMEM (Thermo Fisher Scientific) supplemented with 2% FCS and 10% healthy control or patient plasma (after inactivation at 56 °C, for 20 minutes) were either left unstimulated or were stimulated with IFN-α2 (reference number 130–108-984; Miltenyi Biotec) or IFN-ω (reference number SRP3061; Merck) at 10 ng/ml for 16 hours at 37 °C. Each sample was tested in duplicate for each cytokine. This concentration was chosen due to its higher sensitivity in detecting clinically relevant neutralizing activity, especially in critically ill patients. Finally, cells were lysed for 20 minutes at room temperature, and luciferase levels were measured with the Dual-Luciferase Reporter 1000 assay system (reference number E1980; Promega) according to the manufacturer’s protocol. Luminescence intensity was measured with a FlexStation 3 multi-mode microplate reader (Molecular Devices, Sunnyvale, CA, USA). Firefly luciferase activity values were normalized against Renilla luciferase activity values. These values were then normalized against the median level of induction for non-neutralizing samples and expressed as a percentage. Samples were considered neutralizing if luciferase induction after normalization against Renilla luciferase activity was below 15% the median value for controls tested the same day.

### Quantification of inflammatory markers

Protein levels were quantified in serum samples using the Cytokine Storm 21-Plex Human ProcartaPlex™ Panel (Thermo Fisher Scientific, Waltham, MA, USA; reference number: EPX210–15850-901), a Luminex xMAP technology-based multiplex assay that utilizes magnetic beads. The kit enables exploration of immune function in hyperinflammation by analyzing 21 cytokines and chemokines, including: G-CSF, GM-CSF, IFN-α, IFN-γ, IL-1β, IL-2, IL-4, IL-5, IL-6, IL-8, IL-10, IL-12p70, IL-13, IL-17A, IL-18, TNF-α, TNF-β, IP-10 (CXCL10), MCP-1 (CCL2), MIP-1α (CCL3), and MIP-1β (CCL4). The experiments were performed according to the manufacturer’s instructions. The analyses were performed on a Luminex 200 system, and **median fluorescence intensity (MFI) was used for data presentation and comparison between groups, as the main objective was to perform a relative quantification of cytokine levels.**

### Statistical analysis

Statistical analyses were performed using JASP software (version 0.18.0.1.0). Graphs were generated with GraphPad Prism (version 9.5.0). The Shapiro–Wilk test was applied to assess the normality of continuous variables. Depending on the distribution, group comparisons were conducted using either Student’s t test or the Mann–Whitney U test. Pearson’s chi-squared or Fisher’s exact tests were employed for categorical variables when indicated. Categorical data were presented as absolute frequencies and percentages, while continuous variables were reported as medians with interquartile ranges. The Kaplan–Meier method was used to estimate the influence of auto-Abs on the risk of death. Time was considered from hospital admission to outcome. The log-rank test was used to compare the survival curves. To assess the independent impact of auto-Abs on survival while accounting for potential confounding factors, a Cox proportional hazards regression analysis was performed. A p-value of < 0.05 was considered statistically significant.

## Supplementary Material

Supplementary Material

Online supplemental material

Fig. S1 shows serum levels of cytokines among patients positive or negative for autoantibodies against type I IFNs. Table S1 presents the multivariate Cox proportional hazards regression analysis for predictors of mortality in critically ill COVID-19 patients.

## Figures and Tables

**Table 1.** Baseline characteristics of patients included in the study, stratified by presence of anti-IFN-α2 and/or anti-IFN-ω autoantibodies.

Variables	Total n= 209	Positive n= 14	Negative n= 195	*p-*value
Age, median (IQR)	57 (45–70)	75 (58–80)	56 (44–67)	< 0.001
Sex				
Male, n (%)	130 (62.2)	10 (71.4)	120 (61.5)	0.461
Female, n (%)	79 (37.8)	4 (28.6)	75 (38.5)	
Comorbidities				
Hypertension, n (%)	117 (55.9)	6 (42.8)	111 (56.9)	0.306
Diabetes mellitus, n (%)	75 (35.8)	4 (28.5)	71 (36.4)	0.555
Obesity, n (%)	56 (26.7)	1 (7.1)	55 (28.2)	0.086
Chronic kidney disease, n (%)	17 (8.1)	3 (21.4)	14 (7.1)	0.060
Chronic heart disease, n (%)	11 (5.2)	4 (28.5)	7 (3.5)	< 0.001
COPD, n (%)	9 (4.3)	1 (7.1)	8 (4.1)	0.588
Cancer, n (%)	4 (1.9)	0	4 (2.0)	0.588

Continuous variables were compared using the t-Student test. Categorical variables were compared using the chi-square test or Fisher’s exact test when appropriate. IQR: Interquartile range, COPD: chronic obstructive pulmonary disease.

**Table 2.** Laboratory data from hospital admission of critically ill patients according to positivity for anti-IFN-α2 and/or anti-IFN-ω autoantibodies.

Variables	Total	Positive	Negative	p-value
Glycemia, mg/dL; median (IQR)	166 (131–248)	163 (135–250)	167 (130–247)	0.831
Total leukocytes, counts/mm^3^; median (IQR)	10,470 (10,470–13,290)	12,300 (10,532–16,547)	10,400 (7,745–13,260)	0.136
Neutrophils, counts/mm^3^; median (IQR)	8,784 (6,545–11,694)	10,677 (9,288–15,052)	8,665 (6,476–11,680)	0.086
Lymphocytes, counts/mm^3^; median (IQR)	844 (595–1190)	874 (767–1103)	822 (576–1196)	0.541
Monocytes, counts/mm^3^; median (IQR)	536 (341–818)	492 (460–586)	546 (334–845)	0.812
Platelets, counts/mm^3^; median (IQR)	275,000 (201,500–340,000)	271,000 (172,000–338,250)	275,000 (204,000–340,000)	0.893
aPTT, seconds; median (IQR)	29.20 (24.90–32.45)	27.90 (25.45–31.30)	29.25 (24.87–32.95)	0.574
AST, U/L; median (IQR)	47.30 (31.20–68.20)	37.80 (29.85–51.70)	48.15 (31.37–70.75)	0.409
ALT, U/L; median (IQR)	34.60 (24.30–61.10)	30.20 (22.60–56.80)	35.60 (24.47–64.87)	0.533
Total Bilirrubin, mg/dL; median (IQR)	0.34 (0.23–0.53)	0.30 (0.24–0.38)	0.34 (0.23–0.54)	0.719
Creatinine, mg/dL; median (IQR)	0.90 (0.60–1.30)	1.10 (0.90–1.37)	0.90 (0.60–1.30)	0.106
CRP, mg/L; median (IQR)	130.35 (65.02–255.17)	172.80 (92.90–360.00)	130.30 (64.10–240.80)	0.217
D-Dimer, mg/L; median (IQR)	1.80 (0.80–5.50)	2.26 (0.91–3.95)	1.80 (0.80–5.65)	0.792
Urea, mg/dL; median (IQR)	43.80 (30.55–71.70)	53.75 (33.25–62.25)	42.70 (30.65–73.70)	0.548

Results are shown as median and interquartile range (IQR). Mann-Whitney test was used for comparison between groups. aPTT: activated partial thromboplastin time, AST: aspartate aminotransferase, ALT: alanine aminotransferase, CRP: C-reactive protein.

**Table 3.** Length of stay, complications and outcome of critically ill patients according positivity for anti-IFN-α2 and/or anti-IFN-ω autoantibodies.

Variables	Total	Positive	Negative	*p*-value
Lenght of ICU stay (days), median (IQR)	13 (6–23)	10 (8–12)	13 (6–23)	0,163
Invasive ventilatory support, n (%)	171 (81.8)	13 (92.8)	158 (81.0)	0.268
Hemodialysis, n (%)	59 (29.0)	7 (50.0)	52 (27.5)	0.074
Sepsis, n (%)	44 (21.0)	4 (28.5)	40 (20.5)	0.475
Shock, n (%)	61 (29.1)	5 (35.7)	56 (28.7)	0.578
Heart failure, n (%)	7 (3.3)	1 (7.1)	6 (3.0)	0.414
Death, n (%)	85 (40.8)	8 (57.1)	77 (39.6)	0.200

Continuous variables were compared using the Mann-Whitney test. Categorical variables were compared using the chi-square test or Fisher’s exact test when appropriate. ICU: intensive care unit, IQR: interquartile range.

## Data Availability

The datasets used and/or analyzed during the current study are available from the corresponding author on reasonable request.
