# Supplementary material for: Prevalence and association of type I IFN autoantibodies with clinical outcomes in critically ill Brazilian COVID-19 patients
Source: J Hum Immun. 2025 Jul 8;1(3):e20250031. doi: 10.70962/jhi.20250031 (PMC12700596; doi:10.70962/jhi.20250031)
Supplement: Table S1 — presents the multivariate Cox proportional hazards regression analysis for predictors of mortality in critically ill COVID-19 patients. [file jhi_20250031_tables1.pdf]

Table S1. Multivariate Cox proportional hazards regression analysis for predictors of mortality in critically ill COVID-19 patients.

**Variable**	***p*-value**	**HR**	**CI (95%)**
Age (years)	0.042	1.01	1.00-1.02
Chronic heart disease	0.003	3.47	1.51-7.94
Type I IFN auto-Abs	0.350	1.47	0.65-3.33

HR: hazard ratio
