# Supplementary material for: Prevalence and association of type I IFN autoantibodies with clinical outcomes in critically ill Brazilian COVID-19 patients
Source: J Hum Immun. Author manuscript; Available in PMC 2025 Dec 13. (PMC12700596; doi:10.70962/jhi.20250031)
Supplement: Supplementary file 1 [file NIHMS2116767-supplement-Supplementary_Material.pdf]

Supplementary Material

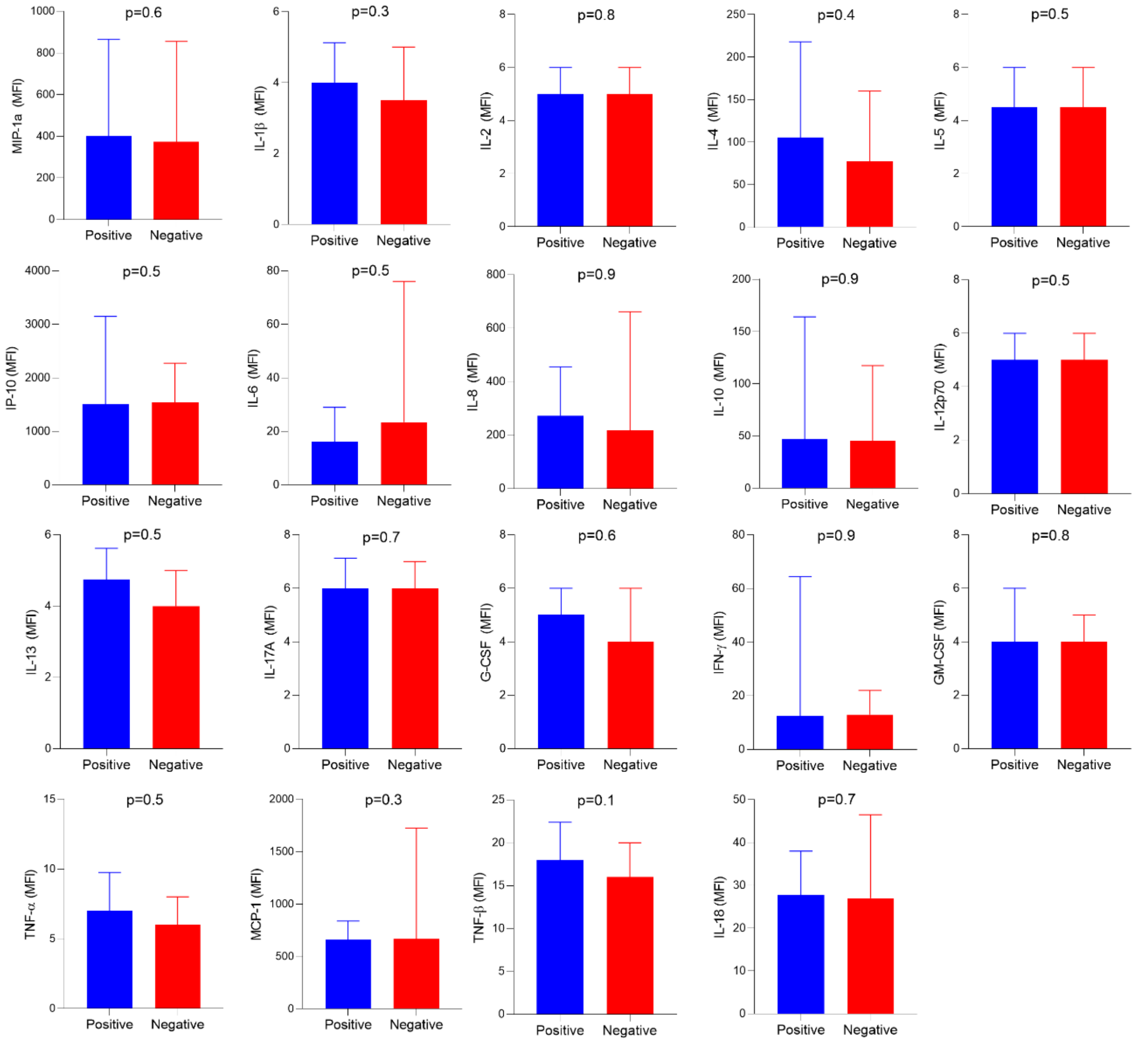

Figure S1. Serum levels of cytokines among patients positive or negative for autoantibodies against type I IFNs. Data are shown as median with interquartile range. The Mann–Whitney test was used to compare the groups. IFN: interferon; MFI: mean fluorescence intensity.

Table S1. Multivariate Cox proportional hazards regression analysis for predictors of mortality in critically ill COVID-19 patients.

Variable	*p*-value	HR	CI (95%)
Age (years)	0.042	1.01	1.00-1.02
Chronic heart disease	0.003	3.47	1.51-7.94
Type I IFN auto-Abs	0.350	1.47	0.65-3.33

HR: hazard ratio
